# Obesity, Weight Loss, Lifestyle Interventions, and Autosomal Dominant Polycystic Kidney Disease

**DOI:** 10.3390/kidneydial2010013

**Published:** 2022-03-04

**Authors:** Cortney Steele, Kristen Nowak

**Affiliations:** Division of Renal Diseases and Hypertension, Anschutz Medical Campus, University of Colorado, Aurora, CO 80045, USA

**Keywords:** adipose tissue, chronic kidney disease, weight loss

## Abstract

Obesity remains a growing public health concern in industrialized countries around the world. The prevalence of obesity has also continued to rise in those with chronic kidney disease. Epidemiological data suggests those with overweight and obesity, measured by body mass index, have an increased risk for rapid kidney disease progression. Autosomal dominant polycystic kidney disease causes growth and proliferation of kidney cysts resulting in a reduction in kidney function in the majority of adults. An accumulation of adipose tissue may further exacerbate the metabolic defects that have been associated with ADPKD by affecting various cell signaling pathways. Lifestyle interventions inducing weight loss might help delay disease progression by reducing adipose tissue and systematic inflammation. Further research is needed to determine the mechanistic influence of adipose tissue on disease progression.

## 1. Introduction

The definition of obesity simplified is excessive body weight relative to height; however, the phenotype draws complex alterations metabolically and has detrimental effects on an individual’s overall health [1]. The World Health Organization (WHO), International Obesity Task Force (IOTF), among other reputable organizations, recognize that for those ≥20 years of age, a body mass index (BMI) ranging from 18.50 to 24.99 kg/m^2^ is normal, ≥25.00 to 29.99 kg/m^2^ is overweight, and ≥30.00 kg/m^2^ is obese [2].

The prevalence of obesity continues to rise and is a major public health concern in industrialized countries globally [3–5]. Individuals with obesity are at risk for developing comorbid conditions including cardiovascular disease (CVD), hypertension, gastrointestinal disorders, chronic kidney disease (CKD), type 2 diabetes (T2D), as well as other diseases that may indirectly increase mortality risk [6,7].

Obesity indirectly causes strain on the kidneys by increasing blood pressure, intensifying renal tubular sodium reabsorption, and weakening pressure natriuresis [8–10]. These events lead to volume expansion by stimulation of the sympathetic nervous system and the renin-angiotensin-aldosterone system (RAAS) [11–14]. Physical compression of the kidneys from surplus visceral adipose tissue also impacts kidney health and function [11,15,16]. Obesity also can lead to renal vasodilation and glomerular hyperfiltration that initially serve as compensatory mechanisms to maintain a sodium balance in the face of increased tubular reabsorption [17]. These potential mechanisms may make obesity a risk for the development and progression of chronic kidney disease (CKD) [11,17–20].

Similar to the general population, evidence suggests obesity is on the rise in those with CKD [21]. The average BMI from 1995 to 2002 has increased from 25.7 to 27.5 kg/m^2^ in patients with end-stage kidney disease (ESKD) [22]. The incidence of total obesity and obesity stage 2 (BMI and >35 kg/m^2^) increased by 33 and 63%, respectively, in this ESKD cohort [22]. In a large cohort study consisting of 3334 CKD patients from Queensland, Australia in 2011, 18.9% had a normal BMI (18.5–24.9 kg/m^2^), 29.9% were overweight (BMI 25–29.9 kg/m^2^), 25.1% were mildly obese (BMI 30–34.9 kg/m^2^), and 26.1% were moderately obese+ (BMI ≥ 35 kg/m^2^). Thus, in this cohort, 81.1% of the CKD patients were either overweight or obese [23].

**Figure 1.**
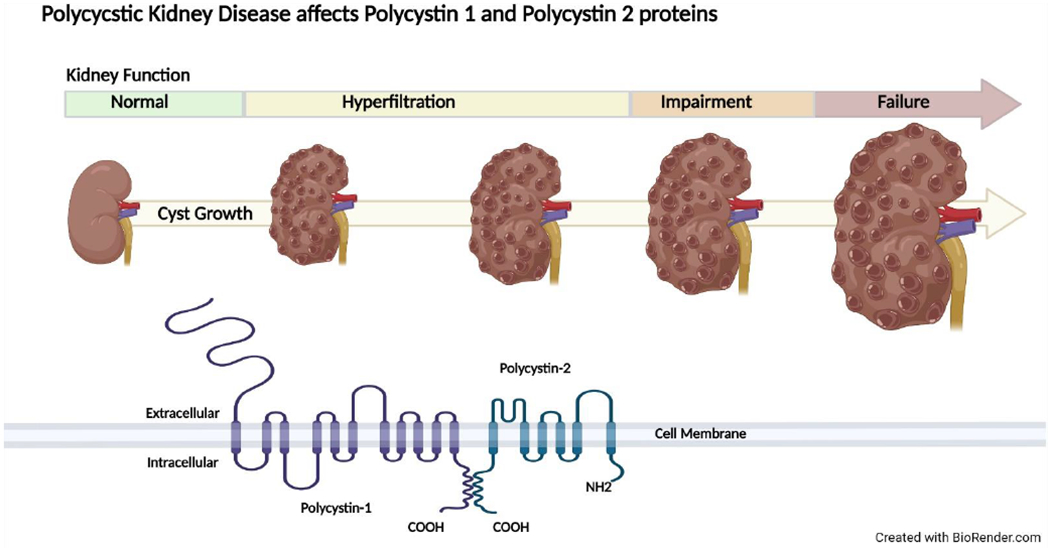
Autosomal polycystic kidney disease (ADPKD) progression and genetic mutations. ADPKD increases total kidney volume from the initiation and proliferation of kidney cysts, causing reductions in kidney function leading to end-stage kidney disease. Polycystin 1 and Polycystin 2 proteins are affected in ADPKD.

Polycystic kidney disease (PKD), a form of CKD, is the most common genetic cause of kidney failure affecting >10 million people worldwide [24]. The genetic disorder is characterized by progressive development and enlargement of multiple renal cysts that ultimately lead to loss of kidney function in the majority of afflicted patients [25]. Autosomal dominant PKD (ADPKD) is the most common form of PKD and is primarily caused by mutations in the *PKD1* and *PKD2* genes, encoding the polycystin 1 and polycystin 2 proteins (Figure 1) [24]. Decreases in kidney function typically do not occur until adulthood in those with ADPKD [24]. The prevalence of overweight and obesity has also increased in the ADPKD community.

In the HALT Progression of Polycystic Kidney Disease Study A (HALT-PKD Study A), which included individuals in the United States averaging 37 years of age with early-stage ADPKD, 62.7% of participants were overweight or obese [26,27]. The average BMI in this trial was 27.1 kg/m^2^ [26,27]. The prevalence of overweight and obesity continue to rise, impacting many populations, including those with CKD and ADPKD.

This narrative review will summarize supporting data in humans in the general population, CKD population, and ADPKD population in relation to obesity. We will explore the potential mechanistic evidence related to the role of adipose tissue in kidney disease. Finally, we will discuss the potential effects of lifestyle modifications that have been studied or are currently under investigation.

## 2. Epidemiological Data on BMI and Kidney Disease

### 2.1. General Population

Obesity increases the risk for kidney disease in the general population [28]. Obesity classified by BMI, increased waist circumference, and increased visceral adipose tissue are associated with elevated albuminuria in the general population [29–32]. After adjusting for potential confounders, higher BMI is associated with increased risk of CKD [18,33,34], reduced estimated glomerular filtration rate (eGFR) [35], decline in kidney function (eGFR slope) [36], and incidence of ESKD [19,20,37] in the general population. A higher BMI at the baseline and increases in BMI over 14 years are associated with a greater risk of CKD [33]. Larger waist circumference is also associated with ESKD, even after adjustment for BMI [22]. Pinto-Sietsma et al. found lean, overweight, and obese subjects with central fat distribution were all at risk for diminished filtration [29]. Central fat distribution has been defined as a waist-hip ratio of ≥0.9 for men and ≥0.8 for women [29,38]. Collectively, this evidence supports that fat distribution may elevate the risk for kidney disease even more than BMI.

### 2.2. Chronic Kidney Disease

A greater prevalence of overweight and obesity has been observed in males aged 45–64 years with CKD stages 3b and 4 (≤44 mL/min/1.73 m^2^) when compared to females and to CKD stages 1–3a (≥45 mL/min/1.73 m^2^) [39]. Higher BMI is associated with increased risk for incident of CKD and advanced CKD (Stage 4–5; eGFR ≤ 29 mL/min/1.73 m^2^) [18,33,34,40]. A male-sex-specific association between increased BMI and CKD has been noted in several studies [19,39,41,42]. Notably, BMI may reflect visceral fat more effectively in males when compared to females [43,44]. In addition, the occurrence of CKD progression, as measured by the rate of eGFR decline per year (>1 mL/min/1.73 m^2^/year), was greater in those with overweight and obese when compared to normal-weight CKD patients [45].

However, there have been several studies that have found no association between BMI and progression of disease in individuals with CKD [23,46–49]. Commonly referred to as the “obesity paradox”, epidemiological studies have demonstrated a lower relative risk of death in patients who are overweight or obese with ESKD, stroke, and heart failure, among other conditions [50–56]. Specifically, dialysis patients who are overweight or obese have a decreased mortality risk [53]. Lu et al. identified a U-shaped association between BMI and risk of kidney disease progression in a large cohort of United States Veterans, demonstrating that those with overweight and mild obesity (BMI 25–35 kg/m^2^) had more favorable clinical outcomes [36]. This phenomenon might be explained by the inherent complexity of chronic diseases, unmeasured risk factors, or bias related to participant selection. There are constraints to using BMI in that it does not account for muscle mass, peripheral and abdominal adipose tissue mass, and bone; consequently, the results should be considered under these limitations [36]. However, globally BMI is the primary measurement to evaluate and define obesity [2]. Molnar et al. found hemodialysis patients with lower BMI or muscle mass and/or unintentional weight or muscle loss had higher mortality [54]. Interestingly, the waist to hip ratio and waist circumference, but not BMI, was associated with mortality in a cohort of patients with CKD and ESKD [43,44]. Additionally, those with CKD who were in the normal BMI category with central obesity had an increased risk of coronary artery calcification [57]. Collectively, these observations again underscore that central obesity might be key in disease development and progression.

### 2.3. Autosomal Dominant Polycystic Kidney Disease

Distinct from other etiologies of CKD, total kidney volume (TKV), often adjusted by height, has been identified as the best biomarker for ADPKD progression, particularly in early-stage disease [58,59]. In addition, Mayo imaging classification helps predict loss of kidney function based on TKV, age, height, and sex [59]. HALT-PKD Study A was a randomized, double-blind, placebo-controlled study in non-diabetic patients with early-stage ADPKD [26]. In HALT-PKD study A, baseline BMI was significantly associated with baseline height-adjusted total kidney volume (htTKV) in men only [60]. Body-surface area was also an independent predictor of baseline htTKV and baseline eGFR in the HALT Studies [60].

In a fully adjusted model accounting for age, sex, race/ethnicity, group randomization, systolic blood pressure, eGFR, urinary albumin excretion, baseline TKV, baseline liver volume, serum class, and mutation class, a higher BMI was associated with a greater annual percent change in TKV in patients with early-stage ADPKD participating in HALT study A [61]. Obesity was also associated with a faster decline in eGFR [61]. These results were subsequently confirmed in individuals with early-stage ADPKD participating in the Tolvaptan Efficacy and Safety in the Management of Autosomal Dominant Polycystic Kidney Disease and Its Outcomes (TEMPO 3–4) trial. After adjustment for age, sex, race/ethnicity, group randomization, systolic blood pressure, serum glucose, baseline eGFR, urinary microalbumin, plasma copeptin, and mutation class, a higher BMI was again associated with a greater annual change in TKV [27]. Notably, the efficacy of tolvaptan was independent of BMI [27]. Of importance, in those with ADPKD, enlarged kidneys may contribute considerably to overall body weight, which may impact BMI calculations, expanding the limitations of BMI discussed previously [62]. However, in the analyses from HALT study A and TEMPO 3:4, BMI was calculated after subtracting the contribution of the kidneys to total body weight, thus controlling for this factor.

The epidemiological data present a strong case overall that overweight and obesity, as measured by BMI, may increase the risk of incident kidney disease and progression, although these studies are observational and there are inherent limitations. Specifically, the ADPKD studies present strong evidence that overweight and obesity are risk factors for disease progression measured by TKV. The mixed evidence in the CKD population, particularly involving the association of BMI with eGFR, may be due to the inability to account for body composition, fat distribution, and other unmeasured risk factors in the CKD population. This underscores the importance that central body fatness is a stronger predictor of overall disease risk when compared to overall body size [63,64].

## 3. Potential Role of Adipose Tissue

Adipose tissue is important in helping to maintain lipid and glucose homeostasis [65]. However, obesity and increased accumulation of adipose tissue result in a pro-inflammatory, hyperlipidemic, and insulin-resistant environment. This dysfunctional adipose tissue can contribute to type 2 diabetes and promote cardiovascular disease [66]. Excessive adipose tissue has the potential to be even more harmful to individuals with ADPKD because of the known metabolic alterations linked to the genetic disease [67].

### 3.1. Types and Distribution of Adipose Tissue

Obesity increases total adipose tissue. When in excess, adipose tissue becomes displaced, producing fat deposits not only surrounding the kidneys but also other vital organs. Visceral adipose tissue is hormonally active and has distinct biochemical attributes that impact various normal and pathological processes in the human body [68]. In humans, the only measurements that can produce direct measures of cross-sectional areas or volumetric measures of visceral adipose tissue are through a computerized tomography (CT) scan or magnetic resonance imaging [68]. Visceral adipose tissue is metabolically active and stimulates the release of fatty acids into circulation. Accumulation of visceral fat or visceral obesity leads to a cascade of negative events that promote metabolic syndrome including hyperinsulinemia, systematic inflammation, and dyslipidemia [69]. Both increases in visceral and subcutaneous adipose tissue were associated with a decrease in eGFR (cystatin-based equation) in the individuals from the Framingham Offspring Study (general population) who underwent abdominal CT scans [70]. The location of the adipose tissue, as well as the type of adipose tissue, determines the impact it will have on biochemical processes.

Adipose tissue can be classified into three subsets: white, brown, and beige adipose tissue. About eighty percent of adipose tissue found in lean healthy subjects is subcutaneous white adipose tissue (WAT) [71]. In humans, brown fat found near the regions of the spine accounts for about 2% of total fat [72]. Beige adipose tissue comprises both white and brown adipose tissue. Genetics may also influence WAT distribution [72]. Recently, Fas Binding Factor 1 (FBF1) deficiency has been shown to stimulate beiging and beneficial growth of WAT [73]. Interestingly, FBF1 controlled the beiging program via a cilia-specific, A-kinase anchoring protein (AKAP9)-dependent, protein kinase A (PKA) signaling, supporting a central role for primary cilia in the fate determination of preadipocytes and the generation of metabolically healthy adipose tissue [73].

In those without obesity, WAT is a vital energy source in that it acts as a lipid storage reserve. However, obesity causes WAT to become metabolically dysfunctional [74]. Perinephric adipose tissue (PAT) is a type of WAT that encircles the kidney and supports kidney function [75]. In the Framingham Heart Study, individuals with higher PAT had a higher risk of hypertension, even with adjustment for BMI and visceral fat [76]. During tumorigenesis of clear cell renal cell carcinoma (ccRCC), cells preferentially invade PAT, a process associated with poor prognosis. The cells secrete a parathyroid-hormone-related protein (PTHrP), which promotes the browning of PAT by PKA activation, and the excess release of lactate mediated by thermogenesis, enhancing ccRCC growth. Inhibiting the ccRCC-adipocyte feedback prevents cell growth, invasion, and metastasis [77]. This evidence could potentially be applied to an ADPKD model to suppress the proliferation of kidney cysts.

### 3.2. Harmful Effects of Adipose Tissue

**Figure 2.**
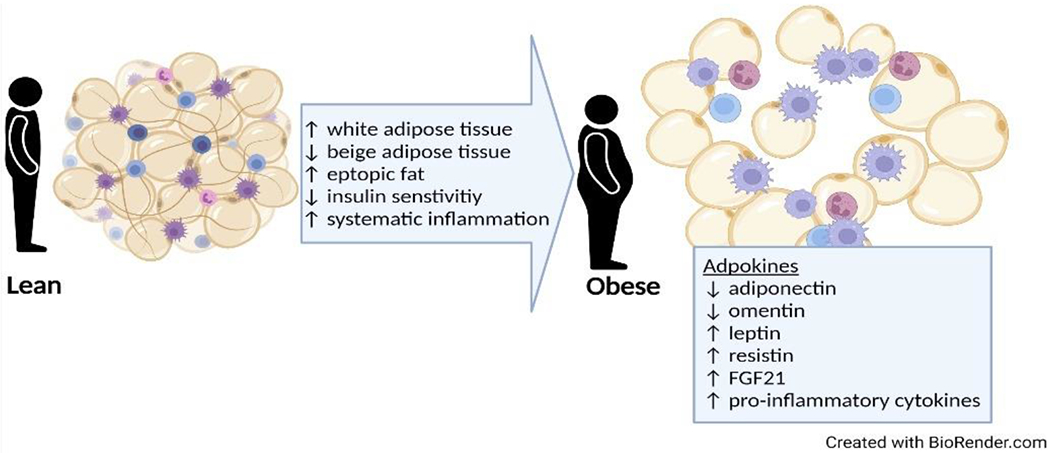
Harmful effects of increased adipose tissue with weight gain. With weight gain there is an increase in total adipose tissue (white and brown adipose tissue); excessive accumulation of adipose tissue causes insulin sensitivity and systematic inflammation. The increase in adipokines leads to altered hormone secretion and pro-inflammatory cytokine production. Abbreviation: FGF21, fibroblast growth factor 2.

Adipose tissue releases adipokines, growth factors, pro-inflammatory cytokines, and chemotactic cytokines [78]. Adipokines secrete hormones such as leptin [79], omentin [80], adiponectin [81], resistin [82,83], and fibroblast growth factor 21 [84], which are influenced by weight gain and increased adipose tissue accumulation (Figure 2).

Evidence suggests that the elevations in pro-inflammatory cytokines observed in obesity, including interleukin 6 (IL-6), tumor necrosis factor-alpha (TNF-α), monocyte chemoattractant protein-1 (MCP-1), and serum amyloid, can be reduced via weight loss [85–88]. Epicardial adipose tissue thickness measured via echocardiography was independently associated with highly sensitive C-reactive protein (hs-CRP) concentrations in normotensive ADPKD patients with preserved renal function [89]. In general, reductions in adipose tissue in individuals with ADPKD who are overweight and obese could potentially induce a shift in cytokines and hormones, reducing the effects of ADPKD-associated metabolic dysfunction.

## 4. Pathways Relevant to Obesity and ADPKD

Several signaling pathways promote inflammation and influence cystogenesis, including the c-Jun N-terminal kinase and the inhibitor of kappa B kinase beta-nuclear factor-kappa B pathways [90]. In addition, many hormones and cytokines altered with obesity may influence ADPKD progression, including increased levels of insulin, insulin-like growth factor 1 (IGF-1), leptin, TNF-α, and IL-6, as well as decreased adiponectin [85–88]. These alterations can promote an increase in the activity of the phosphatidylinositol 3-kinase (PI3K)/Akt signal pathway, which impacts cell survival and growth [91].

**Figure 3.**
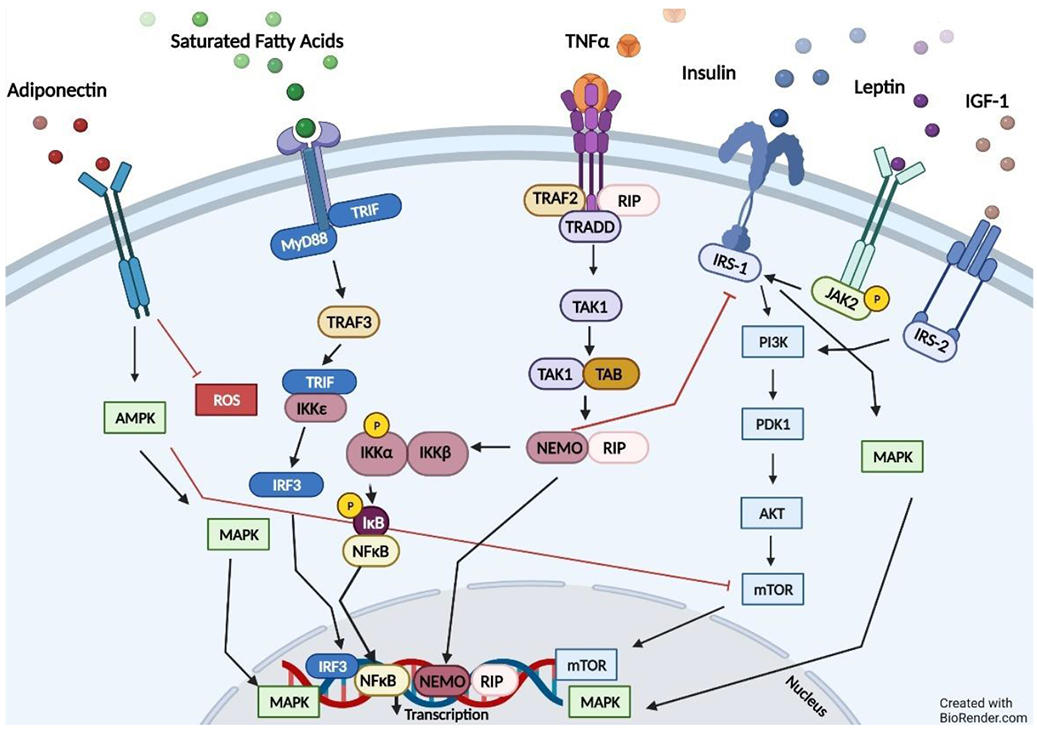
Signaling pathways impacted by obesity. Obesity leads to the activation of inflammatory signaling pathways in metabolic cells through several pathways. The increase in pro-inflammatory cytokines can then lead to intensified receptor activation as the cytokine signals combine with excess nutrients, especially fatty acids. (Abbreviations: AMPK, AMP-activated protein kinase; MAPK, mitogen-activated protein kinase; ROS, reactive oxygen species; MyD88, myeloid differentiation primary response 88; TRIF, TIR-domain-containing adapter-inducing interferon-β; TRAF3, TNF receptor associated factor 3; IRF3, interferon regulatory factor 3; IKKϵ, IkappaB kinase ϵ; IKKβ, IkappaB kinase β; IKKα, IkappaB kinase α; IκB, nuclear factor-κB; NFκB, nuclear factor kappa B; TNFα, tumor necrosis factor α; TRAF2, TNF receptor associated factor 2; RIP, receptor-interacting protein; TRADD, tumor necrosis factor receptor type 1 associated death domain protein; TAK1, transforming growth factor-β-activated kinase; TAB1, TAK-1-binding protein; NEMO, NF-κB essential modulator; IRS-1, insulin receptor substrate-1; IRS-2, insulin receptor substrate-2; IGF-1, insulin-like growth factor 1; JAK2, janus-activated kinase 2; PI3K, phosphoinositide 3-kinases; PDK1, pyruvate dehydrogenase kinase 1; AKT, protein kinase B; mTOR, mammalian target of rapamycin).

Both leptin and adiponectin also act through the AMP-activated protein kinase (AMPK) pathway [92]. Studies have demonstrated that adiponectin activates AMPK via adiponectin receptor 1, and AMPK is known to inhibit the mammalian target of rapamycin (mTOR) pathway [93,94]. Cytokines secreted from visceral adipose tissue promote a pro-tumorigenic environment, which may also be applicable to ADPKD. Interleukin-6 (IL-6) can activate the signal transducer and activator of transcription 3 (STAT3) and extracellular signal-regulated kinase (ERK) signaling, which are known to be increased in PKD [95]. TNF-α initiates cell signaling through tumor necrosis factor receptor 1 (TNRF1), activating TNFR1-associated death domain (TRADD), TRN receptor-associated factor-2 (TRAF2), and a receptor-interacting protein (RIP). The pathway leads to an active NF-κB essential modulator (NEMO), which translocates to the nucleus to promote the production of pro-inflammatory genes [96]. Insulin binds to the insulin receptor (IR), activating receptor tyrosine kinase, which allows binding of insulin receptor substrates (IRS). IRS then stimulates cell proliferation via the PI3K-AKT system, the mammalian target of rapamycin (mTOR), and the MAPK systems [97]. Saturated fatty acids (SFAs) can bind to Fetuin-A, which is an endogenous ligand of toll-like receptor 2 (TLR2) or TLR4, and invokes transcription of interferon regulatory factor 3 (IRF3) [98]. SFAs are the main non-esterified fatty acid (NEFA) in the circulation of obese subjects [98]. SFAs activate an inflammatory response via TLR4 signaling, serving as a connection between fatty acid excess and chronic low-grade inflammation [98]. All these signaling pathways play a role in inflammation and cell proliferation (Figure 3).

## 5. Impaired Fatty Oxidation

There is also evidence of impaired fatty acid oxidation in *Pkd1* mutant mice [98]. However, lowering the lipid content in chow modified cystic disease by correcting the fatty acid oxidation impairment in mice [99]. Female mice had a less severe kidney phenotype, which was associated with protection from alterations in lipid metabolism compared to males [98]. In HALT Study A, there was a significant positive association between two large chain triglycerides (Triglyceride(51:3) [M + K]+ and Triglyceride(53:3) [M + K]+) and htTKV at baseline [100]. In the HALT studies, there was also evidence of altered fatty acid metabolism, including lipoxygenase pathways (LOX) [101]. These findings provide evidence that ADPKD may further be exacerbated with obesity from the inability to oxidize fatty acids.

## 6. Weight Loss and Kidney Function

### 6.1. General Population and Other Disease Profiles

In the general population, there have been numerous randomized control trials assessing weight-loss interventions. A recent meta-analysis concluded that weight-reducing diets, generally low in overall fat and saturated fat, with or without exercise advice or programs, have the potential to reduce premature all-cause mortality in adults with obesity [102]. In adults with type 2 diabetes, a weight loss of >5% appears essential for favorable effects on HbA1c, lipids, and blood pressure [103]. In a prospective 3-year cohort study, rapid weight loss was correlated with reduced kidney function measured by eGFR in normal-weight, healthy, non-diabetic males, but improved kidney function in males who were overweight [104].

### 6.2. Chronic Kidney Disease

A systematic review consisting of five controlled and eight uncontrolled trials concluded that weight loss is associated with decreased proteinuria and microalbuminuria in those with CKD [105]. Mechanisms of how weight loss via diet, physical activity, or pharmaceuticals reduces proteinuria may include enhanced blood pressure control, an improved lipid profile, increased insulin sensitivity, reduced leptin concentrations, reduced glomerular hyperfiltration, diminished RAAS activation, and an overall decrease in inflammatory and oxidative stress markers/pathways [106]. In another systematic review evaluating weight loss and kidney function in CKD patients, weight loss, especially via surgical interventions, improved proteinuria, albuminuria, and normalized GFR [107]. Lifestyle interventions completed in obese CKD patients have included primarily caloric restriction diet plans with or without an exercise prescription [108–113]. These lifestyle interventional studies reduced BMI, proteinuria, and albuminuria [56–59]. Additionally, a third recent systematic review concluded that non-surgical weight-loss interventions are effective in reducing body weight and LDL cholesterol in overweight and obese adults with CKD [114]. Overall, lifestyle interventions that invoke weight loss appear to have beneficial effects in adults with CKD; however, more research is needed, including larger and longer trials evaluating the effects of weight loss on CKD progression.

### 6.3. Autosomal Dominant Polycystic Kidney Disease

**Figure 4.**
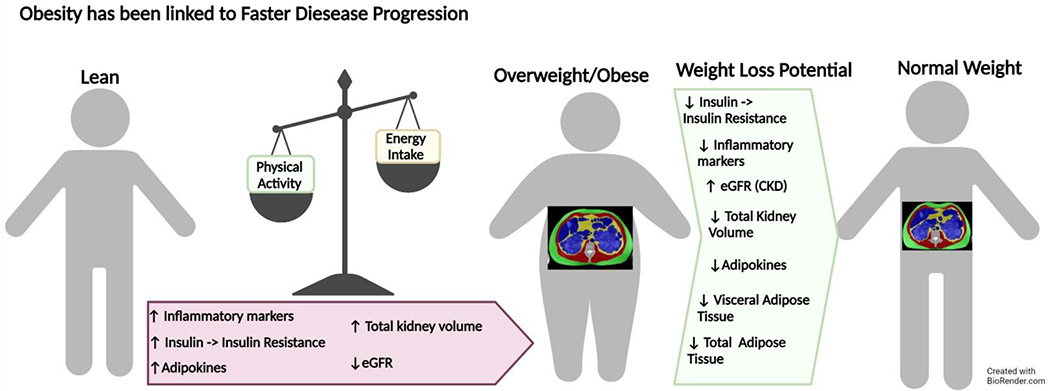
Weight loss interventions may help slow ADPKD disease progression. Obesity has been shown to have negative consequences on disease progression in those with ADPKD. Weight loss may have the potential to reduce total kidney volume, total adiposity, and metabolic and pro-inflammatory responses.

The kidneys rely primarily on fatty acid oxidation (FAO) via aerobic glycolysis to produce adenosine triphosphate and have high metabolic demand, especially in the proximal tubule. A defect in fatty acid oxidation can lead to deleterious effects on the kidneys [115]. Metabolic reprogramming, including altered substrate metabolism, compromised autophagy, and mitochondrial impairment in ADPKD, have been reviewed previously [67]. The majority of pathways affected by ADPKD also have extrarenal implications, making targeted drug therapy more of a challenge [116]. However, altered cellular pathways associated with obesity in individuals with ADPKD could potentially be targeted via lifestyle interventions that induce weight loss as a potential avenue to improve metabolic health (Figure 4).

## 7. Weight Loss Interventions and Kidney Function in ADPKD

### 7.1. Physical Activity Interventions

Collectively, large epidemiological studies and several randomized controlled trials, including both the general population and those with CKD, provide evidence that increased physical activity can reduce the risk of mortality [117–120]. The recommendations for frequency, intensity, time, and type of exercise are still being developed for those with CKD [121]. General recommendations listed for the CKD population include gradually increasing physical activity to the following: aerobic exercise 3–5 days/week for 20–60 min, resistance training 2–3 days/week, and flexibility 2–3 days/week [121]. Another recommendation specific to the PKD population has been to avoid hard contact sports, such as rugby or American football, due to the potential of kidney cysts rupturing from impact [122]. Currently, only one ongoing clinical trial (NCT04907799) involves a dietary intervention (30% daily caloric reduction) as well as physical activity prescription (300 min per week of moderate-intensity exercise) in participants with ADPKD. This trial will provide important insight into the feasibility of an intervention focused on physical activity in APDKD.

### 7.2. Dietary Interventions

The dietary interventions proposed and currently being evaluated in humans have included caloric restriction, intermittent fasting, time-restricted eating, and a ketogenic diet (Table 1) [123–126]. The foundation of these studies is based on murine models demonstrating the benefits of caloric restriction [127,128], time-restricted feeding [128,129], and ketogenic diets [129] on PKD progression, as reviewed elsewhere recently [123]. In addition, one retrospective case series including 131 PKD patients assessed the role of ketogenic dietary interventions via survey, questionnaire-based interviews, and retrospective medical data to assess potential beneficial or adverse effects. Although there are major limitations to this observational study, including selection bias, the investigators found patients’ experience with ketogenic diets were overall beneficial, safe, and feasible from the interviews [130]. Only one trial, which included both daily caloric restriction and intermittent fasting, has evaluated abdominal adiposity via magnetic resonance imaging (MRI). After the 1-year interventions, there were significant reductions in abdominal visceral adipose and total adipose tissue [124]. Slowed kidney growth, as measured by the annual change in htTKV, was associated with body weight and visceral adiposity loss regardless of the intervention [128]. These findings suggest fat distribution, specifically central obesity resulting in an accumulation in visceral adipose tissue, may play a crucial role in ADPKD progression. The primary outcome in the ongoing clinical trial evaluating daily caloric restriction (NCT04907799) is change in htTKV and is currently the largest on-going dietary interventional study in those ADPKD. This trial will also provide insight into the role of change in visceral and subcutaneous adipose tissue in ADPKD progression.

## 8. Novel Future Direction and Clinical Implications

Drug development directly targeting cyst growth to delay the progression of ADPKD remains challenging. Overweight and obesity in those with ADPKD may promote further metabolic dysfunction, accelerating disease progression. Interventions that invoke weight loss and reduce adipose tissue are of interest to prevent or reverse the metabolic consequences of obesity in those with ADPKD. Mechanistic studies evaluating the role of adipose tissue in disease progression are needed to develop targeted treatments and solutions to this increasing health concern.

## Figures and Tables

**Table 1.** Dietary interventions, weight loss, and outcomes in those with ADPKD.

Intervention	Study Design	Length (*n*)	Baseline BMI (kg/m^2^) or Inclusion	Weight Loss	Kidney Outcomes
Caloric Restriction (34% reduction in caloric intake, NCT03342742) Status: Complete [124]	Randomized, double blind, parallel assignment, two experimental arms	12-months (*n* = 15)	34.6 ± 5.1	3-months: −7.1 ± 4.2% 12-months: −9.1 ± 6.0%	Annual % change in htTKV was highly correlated with % change in weight (r = 0.68, *p* = 0.001) and change in BMI at 12-months (r = 0.63, *p* < 0.01)
Intermittent Fasting (20% reduction on three non-consecutive days per week, NCT03342742) Status: Complete [124]	Randomized, double blind, parallel assignment two experimental arms	12-months (*n* = 13)	34.8 ± 5.1	3-months: −5.5 ± 3.3% 12-months: −4.9 ± 5.6%	
Ketogenic Diet (lipids 65%, proteins 30%, and carbohydrate 5% total caloric intake, modified Atkins diet) Status: Complete [125]	Single-arm interventional pilot	3-months (*n* = 3)	25.3 ± 1.4	Hypocaloric ketogenic diet invoked weight loss 1–4.2 kg in those who were overweight	eGFR did not change
Caloric Restriction (30% reduction in caloric intake and increased physical activity, NCT04907799) Status: On-going	Randomized, double-blind, parallel assignment one experimental arm, one control arm	24-months (*n* = 126)	25–45	Secondary outcome change in abdominal adiposity	Primary outcome change in htTKV
Time-Restricted Eating (food intake restricted to an 8-h window, NCT04534985). Status: On-going	Randomized, double-blind, parallel assignment one experimental arm, one active comparator	12-months (*n* = 30)	25–45	Secondary outcomes change in body weight, abdominal adiposity, and body composition	Secondary outcome change in htTKV
Ketogenic Diet (High fat, moderate protein, very low carbohydrate <20 g per day, NCT04680780) Status: On-going	Randomized, parallel assignment, two experimental arms, and one control arm	3-months (*n* = 21)	18.6–34.9	Secondary outcome change in BMI	Secondary outcome change in TKV
Water Fasting (water fasting on 3 consecutive days within the first 14 days of each month, NCT04680780) Status: On-going	Randomized, parallel assignment, two experimental arms, and one control arm	3-months (*n* = 21)	18.6–34.9	Secondary outcome change in BMI	Secondary outcome change in TKV
Acute fasting for 72 h or intake of a ketogenic diet for 14 days (NCT04472624) Status: Complete (results not posted)	Non-randomized (participant selected experimental arm), parallel assignment	72 h (fasting) or 14 days (ketogenic diet) (*n* = 10)	18−35	Secondary outcome absolute and relative change in weight	Primary outcome relative difference in TKV immediately before and after the ketonic state [Time Frame: Visit 2: 2–4 Weeks after enrolment; Visit 3: 3–21 days after Visit 2]
Ketogenic Diet [4–6% carbohydrates, 25–30% proteins, and 60–70% lipids; modified Atkins diet], or a balanced normocaloric diet [55–60% carbohydrates, 10–15% proteins, 25–30% lipids]. Status: On-going [126]	Randomized, parallel group, two experimental arms	12 months (*n* = 90)	>20	Caloric intake will be adjusted for participants to remain weight stable	Primary outcome change in TKV

Abbreviations: BMI, body mass index; htTKV, height-adjusted total kidney volume; eGFR, estimated glomerular filtration rate. Data represented by mean ± SD.
